# Reducing Changeover Time Between Surgeries Through Lean Thinking: An Action Research Project

**DOI:** 10.3389/fmed.2022.822964

**Published:** 2022-04-27

**Authors:** Mirjam Amati, Alan Valnegri, Alessandro Bressan, Davide La Regina, Claudio Tassone, Antonio Lo Piccolo, Francesco Mongelli, Andrea Saporito

**Affiliations:** ^1^Information and Process Management/Supportive Area, Ente Ospedaliero Cantonale, Bellinzona, Switzerland; ^2^Hospital Direction, Bellinzona e Valli Regional Hospital, EOC, Bellinzona, Switzerland; ^3^Department of Surgery, Bellinzona e Valli Regional Hospital, EOC, Bellinzona, Switzerland; ^4^Faculty of Biomedical Sciences, University of Lugano, Lugano, Switzerland; ^5^Operating Theatre, Bellinzona e Valli Regional Hospital, EOC, Bellinzona, Switzerland; ^6^Department of Anesthesia, Bellinzona e Valli Regional Hospital, EOC, Bellinzona, Switzerland

**Keywords:** lean thinking, weekly management, changeover time, standardization, inter-professional collaboration, operating room, surgery

## Abstract

**Background:**

Maximizing the utilization of the operating room suite by safely and efficiently changing over patients is an opportunity to deliver more value to patients and be more efficient in the operating suite. Lean Thinking is a concept that focuses on the waste inadvertently generated during organization and development of an activity, which should maximize customer value while minimizing waste. It has been widely applied to increase process efficiency and foster continuous improvement in healthcare and in the operating room environment. The objective of this paper is to provide insight on how healthcare professionals can be engaged in continuous improvement by embracing Lean Thinking and ultimately reducing changeover time between surgeries.

**Methods:**

Using an action research approach, Lean methodology such as Gemba walks, Process Mapping, Root-Cause-Analysis, and the Single Minute Exchange of Dies (SMED) system was applied to understand the causes of variability and wastes concerning changeovers and improve processes in the context of gynecological- and general surgery. Data were collected and analyzed through observations and video recordings. Problem and issue have been raised to management team attention and included in the annual balanced scorecard of the hospital. This initiative has been also made relevant to the team working in the operating suite and related processes before and after the entry of the patient in the operating suite.

**Results:**

Improved patient flow and inter-professional collaboration through standardized and safer work enabled effective parallel processing and allowed the hospital to reduce changeover time between operations by 25% on average, without changes in terms of infrastructure, technology or resources.

**Conclusion:**

Lean thinking allowed the team to re-evaluate how the whole operating suite performs as a system, by starting from a sub-process as changeover. It is fundamental in order to improve further and obtain sustainable results over time, to act on a system level by defining a common goal between all stakeholders supported by a management and leading system such as visual/weekly management, optimizing planning, implementing standard-works to be followed by every associate and guaranteeing the role of the surgeon as process driver who pull performances.

## Background

The continuous growth of healthcare costs is of major concern for the sustainability of public finances in the advanced Countries (1). Healthcare expenditure in Switzerland per capita and as a share of gross domestic product (GDP) is among the highest in the world, (12.3% of GDP in 2017) (2). Although the overall quality of care is very good, this is not true in each individual case. Insufficient quality and inefficient infrastructure lead to additional costs. In light of this, improving quality of healthcare while controlling cost is a key priority for the Swiss Federal Council’s health policy strategy for the period 2020–2030 and hospitals are under constant pressure to find ways to improve efficiency and productivity while providing high-quality healthcare (2). This pressure is particular strong in the surgical services, which represent a significant portion of both revenue and expense of hospitals (3–5).

This issue has become even more important in the context of the coronavirus (COVID-19) pandemic, during which thousands of interventions were postponed as the Swiss Federal Council banned non-emergency interventions from March to April 2020, and hospitals have been confronted with the need to relocate resources to COVID-19 and intensive care units (6).

Maximizing the utilization of the operating room (OR) suite by safely and efficiently changing over the rooms between surgeries is an opportunity to increase safety, quality and productivity while reducing patients’ waiting time for surgery and, as a result, increasing patients’ satisfaction (7).

Many hospitals worldwide and in Switzerland have embraced the power of Lean Thinking (8). It can be defined as the strategy of focusing on the waste inadvertently generated during organization and development of an activity. Lean is a management system and a method of eliminating waste and create more value for patients (9, 10). Reducing changeover times means reducing the time spent on non-adding-value activities, thus creating more time for taking care of patients. As a management system, Lean encompasses different tools that can be applied on a macro level company-wide and others on a micro level to improve specific processes. Specifically, an effective way to reduce changeover time is the Single-Minute Exchange of Die (SMED) methodology, also known as Quick Changeover (5), developed by Shingo (11) in the industry sector. SMED is a system for reducing the time it takes to change a line from running one product to the next one (i.e., in the OR environment it is the time taken after finishing an operation to start a new one) (5, 11). The application of lean thinking concept on a macro-level and SMED on a micro-level is key to improve OR pathways.

The OR suite functions as a “production site” of its own within a hospital, whose rules and processes differ from the rest of the organization (9). Healthcare professionals (HPs) in the OR are confronted with the challenge of balancing the need for process flexibility with clinical requirements and standardized work to improve safety, quality delivery (lead times) and efficiency (12). Although there is a great deal of natural variability among patients, the principal steps of a given procedure are usually consistent and can be therefore standardized (13).

Standardization through the SMED methodology allows to improve the workflow of these major steps and to reduce the unnatural variability, hence the variability that is not dependent on the characteristics neither of the patients nor of the surgery, but on poor processes. Previous research has shown the positive impact of this methodology and operations management on changeover time between surgeries (12, 14–19). The randomized-controlled trial conducted by Mizumoto et al. (18), for example, showed a 58% reduction in mean changeover time.

At the core of Lean Thinking lies the need for engagement from all stakeholders regarding the process to be improved at different levels of the organization. Process improvement initiatives, in order to be effective and sustainable over time, require the engagement and empowerment of the people closest to the work. Hence, the frontline HPs must be part of this process, as they have intimate knowledge of the work. They feel the ownership of their assigned tasks and can best determine which are the opportunities of improvement. Furthermore, these initiatives require administrative engagement and the support of the hospital executive board (20, 21). The fundamental principles of Lean Thinking include continuous improvement and respect for people (8, 20). However, effective inter-professional collaboration and problem solving may be challenging in hospitals which are traditionally segmented in silos, as people may not be organized or trained to see how the system as a whole is working (22).

The aim of this paper was to provide insight on how HPs can be engaged in continuous improvement by embracing Lean Thinking and ultimately reducing changeover time between surgeries.

## Methods

This paper presents an action research (AR) project which implements Lean Thinking and methodology on a macro-level and the SMED system on a micro-level in the context of gynecologic and general surgery in a public hospital located in Canton Ticino, Switzerland. The project focuses on changeover time between surgeries measured from last stich to incision.

The primary end point of this project was the reduction of changeover times for gynecological and general surgery.

This project was approved by the local Executive Board. Ethical committee was waived as data was fully anonymized and used for internal quality check only.

### Setting

The project was conducted at the Bellinzona e Valli Regional Hospital, Bellinzona, Switzerland, a hospital with 1,300 employees, and of these, more than 100 HPs work in the OR suit. The OR suite has five operating rooms, four induction rooms and one recovery room, performing about 7,000 interventions a year. Our institution recognized the gynecologic and general surgery to be *worst-cases* with changeover times of more than 1 h on average (62 min for gynecology and 64 min for general surgery in 2019).

### Design

This research approach was chosen as it is a collaborative and cyclical process – as Lean Thinking suggest – in which researchers and team members work closely together through five phases: (1) Diagnosis; (2) Action Planning; (3) Taking Action; (4) Evaluating; (5) Specifying learning (23, 24). Action research fosters organizational change, inter-professional collaboration and the empowerment of HPs resulting in better quality of care (23).

Each phase of the process was concretized based on Lean Thinking: Gemba walks, Process Mapping and Root-Cause-Analysis were used both in the diagnosis phase and, in the evaluating phase, SMED was used as the key methodology to analyze changeover processes and define the actions and countermeasures to reduce changeover times. The design of the project and the respective methodology for each phase is reported in Table 1.

**TABLE 1:** Theoretical framework and methods.

When	What	How
	Theoretical framework: action research	Methods: lean methodology
April – May 2020	Preparation	Background: literature review – relevance for patients and the hospital. Preliminary data analysis, Kaizen charter, training.
June – July 2020	Diagnosis	Current situation: data analysis, Gemba walks, process mapping, SMED system (incl. video recording and analysis). Problem statement and goal setting: gap IST-SOLL. Root-cause-analysis: Fishbone Diagram.
August – November 2020	Action planning	Countermeasures: SMED system and action plan, simulation, testing and coaching. Planning of implementing “quick wins.” Measures to apply SMED analysis, key responsibilities and deadlines.
December 2020	Taking action	Implementation of the countermeasures – Standard work, Visual management
January – March 2021	Evaluating	Monitoring and continuous improvement: data analysis and weekly management huddle. Impact on changeover time after 1–3 months. Gemba walks and SMED.
	Specifying learning	Final discussion with the inter-professional team: relevant themes.

*SMED, single-minute exchange of die.*

The project was conducted by an inter-professional team, led by the chair of anesthesia, and consisting of one nurse anesthetist, one surgical technician, one housekeeper, one OR administrative secretary, two surgeons and two head nurses representing the gynecology and general surgery specialties. The whole team was coached and supported by three internal Lean facilitators through each phase of the project. The first one was the Lean manager and researcher as part of the CAS in Healthcare Leader Excellence at the University of Bern. The second one was the head of the Information and Process management team, and the third one was the director of the hospital with his extensive Lean experience of more than 25 years of service in the industry.

The project was organized *via* workshops (physical and virtual) over a total of 42 h, from April 2020 to January 2021, a time period that allowed the team to complete the Action Research cycle twice, and to test and adjust the countermeasures in the daily practice.

### Data Collection and Analysis

Data were collected in two ways. First, the team conducted so called “Gemba” walks. The Japanese term *Gemba* refers to “the real place where work is done” (8). Gemba has become both a tool and a process for engaging inter-professional teams in the analysis, planning, implementation and evaluation of changes (25). Gemba walks have become also a mindset for the entire management and project team. They were conducted in the OR suite and the inpatient units. Although the focus was on changeover processes, the team followed the entire patient flow from the inpatient units to the OR to understand the whole process and the perspectives of different HPs. Team members had different observation roles and met after each changeover process to share their insight in a structured way All activities were tracked and timed. The major steps of the process were mapped on the wall, and problems were identified and written on post-it, which were then linked to the process mapped. By conducting Gemba walks, it was possible for the team to ask questions to the HPs involved in the process to better understand what was happening and why, and the impact on changeover times. The second method of data collection involved creating video recordings of the changeover process in the OR. The video recordings were organized and coordinated by a OR nurse who is passionate about video making. The two ways of data collection conducted in parallel allowed the team to analyzed what was happening and why both inside and outside the OR before and during the changeover process.

Data were analyzed *via* the SMED system, developed by Shingo (11) in the industrial sector: “Single-minute” refers to a changeover process (*setup*) performed in less than 10 min, hence a number of minutes expressed in a single digit, as achieved by Shingo. Although this time may not always be literally achievable, the system frequently leads to dramatic reductions in changeover times at low cost (5, 11). The video recordings were processed by the whole team together in a meeting room by performing the following tasks according to the SMED system (5, 11, 15): describing changeover steps, quantifying the time required to perform each step and separating internal steps (Inside Exchange of Die – IED) from external steps (Outside Exchange of Die – OED). IED refers to changeover steps that must be performed when the OR is not in use (i.e., while the procedure on the previous patient finished). OED refers to changeover steps that can be performed in parallel while the prior procedure is ongoing.

The analysis of such steps was the basis to convert internal to external steps (i.e., parallelization of activities) as much as possible (action planning) and to streamline changeover workflow and standardize all the steps (action planning).

## Results

### Diagnosis

**FIGURE 1:**
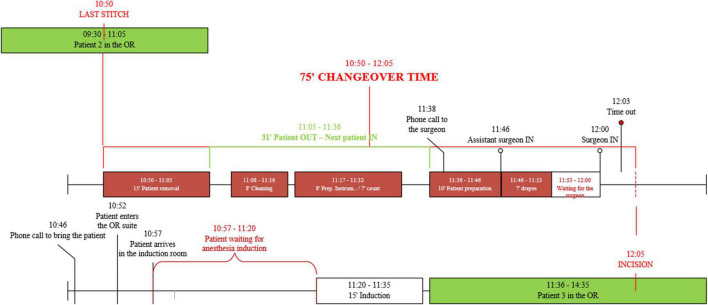
Cause-effect diagram of factors affecting changeover time.

**FIGURE 2:**
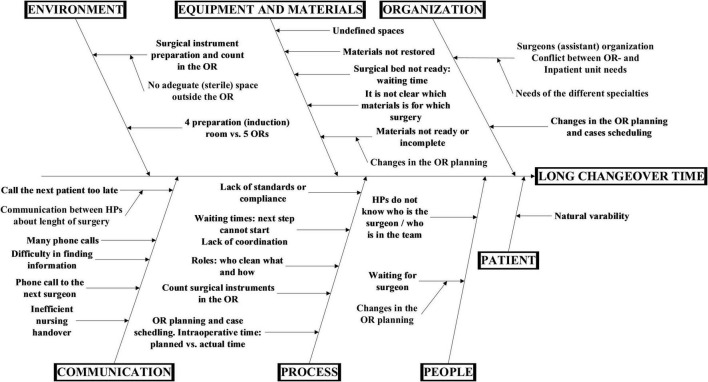
Example of changeover process map.

Gemba walks were conducted over the course of 2 days with the goal of observing three elective surgeries for a total of two changeover times for gynecological and general surgery. A detailed process map is reported in Figure 1. The changeover times for gynecological surgery were 55 to 75 min, consistent with the mean time of the specialty in the hospital, which was 62 min in 2019. Figure 2 shows the cause-effect analysis conducted after the Gemba walks.

The SMED analysis for the first changeover is reported in Table 2. The team started to define the actions and countermeasures in parallel to the analysis. The team concluded that changeover processes could have been done in less than 37 min. Thus, there was a gap of 18 to 38 min for potential improvement.

**TABLE 2:** Single-minute exchange of die (SMED) analysis and countermeasures.

N°	Time	Activity	Roles	Actual duration	Expected duration	Time savings		Countermeasures
						IED	OED	
	0	Last stitch of previous patient						
1		Wound care + waiting for wound drying	Surgical techs 2x	2′30′′	1′35′′	55′′		1. Check manufacturer instructions: glue drying standard (→only a layer of glue with natural drying 95 s is needed). 2. Create standard work on how to restore ORs after surgery for surgeon techs. Define activities that can be parallelized: e.g., remove aspirators, restore instrument table, fluids balance before stitching
2		Remove drapes + patient and wound cleaning	Surgical tech (sterile)	2′26′′	1′26′′	1′		3. Create standard on how to remove drapes and clean the patient with two people (surgical tech and assistant surgeon). 4. To ensure point 2: define the clear role of the assistant surgeon who has to stay in the OR to help the surgical tech
3		Remove patient monitoring (equipment)	Anesthesiologist / nurse anesthetist	19′′	0	19′′		Activities that can be done in parallel with the previous ones. No countermeasure at the moment.
4		Reposition urinary catheter / Prepare patient to leave the OR	Surgical tech (sterile)	1′45′′	15′′	1′30′′	15′′	5. Create standard on how to prepare the patient to leave the OR (respect their privacy and dignity). The reposition of the urinary catheter has to be done in the recovery room (OED step, about 15′′) together with the other activities already defined.
5	+6′	Patient OUT						
6		OR cleaning: trash cans (1′30′′), floor, equipment	Housekeepers 2x	6′ x2	5′ x2	1′		6. Create standard work on how to clean the OR after surgery with one housekeeper and with two housekeepers. Decision: no one can enter the OR until the cleaning process is finished. Define when to call the housekeepers. 7. 5 s methodology to organize space
7		Surgical instruments preparation	Surgical techs 2x	10′ x2	9′	1′		8. Create standard work for the two surgeon techs roles. Standard: patients can be transferred in the OR when the instrument count is starting, see point 9
8		Count surgical instruments and display instrument table	Surgical techs 2x	3′ x2	3′	0		9. Create standard: patients can be transferred in the OR when the instrument count is starting. Here you can save 3 min. This is possible only if the surgeon assistant is responsible for patient positioning. See point 11.
9	+21′	Patient IN						
10		Patient monitoring (equipment)	Anesthesiologist / nurse anesthetist	2′25′′	1′	1′25′′		Activities that can be done in parallel with previous activities. The timing here includes teaching.
11		Patient positioning End of instrument table preparation Assistant surgeon IN	Surgical tech + nurse anesthetist	8′50′′	6′	2′50′′		10. What are the best practice in terms of surgeon organization? To find it out. Certain specialties, e.g., Orthopedics, have an assistant surgeon dedicated to the OR. In General surgery and Gynecological surgery, assistant surgeons work both in the OR and the inpatient units at the same time. 11.1 Define role standard for the assistant surgeon / review actual standard and consolidate it: a) if the assistant surgeon is dedicated to the OR the whole day; b) if the assistant surgeon works in the inpatient units as well and has to be phone called. If b), then define who has to make the call.
12		Skin disinfecting Surgeon IN	Surgical tech (sterile)	2′40′′	2′40′′	0		11.2 Define role standard for the assistant surgeon. Surgeons have to prepare themselves in parallel as so to avoid waiting times. They can so help surgical techs in patient preparation and draping.
13		Draping the patient	Surgical tech (sterile)	2′33′′	2′	33′′		See point 11.
14		Dress assistant surgeon	Surgical tech (sterile) Assistant surgeon	1′21′′	45′′	36′′		11.3 Define role standard for the assistant surgeon: hygienic hand disinfection to be included also in this document. Hands washing has to be done at the entrance of the OR suite; then hygienic hand disinfection as hospital protocol.
15		Prepare surgical instruments + patient positioning Prepare equipment: hysteroscopy equipment, optics, drapes, lights	Surgical tech (sterile)	3′20′′	3′20′′	0		No countermeasure here. See points 12–13.
16	+43′	Time-out Surgeon IN		1′	1′	0		12. Improve Time out process! Protect this time and ensure safety.
17		Preparations for surgery	Surgeons	2′	0	2′		13. The time out must be done immediately prior to the beginning of the procedure and have active involvement of the entire surgical team. Equipment preparation and draping must be done before Time out. Everything must be ready.
18		Preparation for surgery: technical problem	Surgeons	4′40′	0	4′40′′		See point 13.
19	+51′	Incision						
			Total time ∼	54′49′′ 55′	37′1′′ 37′	18′3′′ 18′		The countermeasures would allow to reduce changeover time by 33%, saving 18 min.

#### Internal Steps (IED)

By separating internal steps (IED) from external steps (OED), the analysis showed that internal steps represented the majority of the changeover process, from last stich to incision. These steps included:

1. Patient recovery and preparation for patient to leave the OR, performed by the anesthesiologist/nurse anesthetist and two surgical technicians.
2. Patient leaving the OR (Patient OUT) with the anesthesiologist/nurse anesthetist.
3. Tidying up surgical instruments (two surgical technicians) and OR cleaning (one-two housekeepers).
4. Surgical instruments preparation and counting (two surgical technicians).
5. Patient entering the OR (Patient IN) with the anesthesiologist/nurse anesthetist.
6. Patient preparation and positioning (nurse anesthetist, non-sterile surgical technician).
7. Skin disinfecting and draping (sterile surgical technician).
8. Time-out (all surgical team).
9. Waiting times: patient waiting for induction; OR ready waiting for patient; OR team waiting for surgeons.

#### Conversion of Internal to External Steps

A critical step was converting internal steps to external steps, and the following themes emerged.

(1) Parallelize patient positioning and surgical instruments count

Patient positioning was performed by one surgical technician and the nurse anesthetist, and lasted up to 20 min during which the rest of the team was waiting. Conducting patient positioning in the preparation room, when possible, would allow parallelization of this step with the OR cleaning and / or the surgical instruments preparation and counting. Barriers to this, identified by the team and then addressed as countermeasures, were the absence or passive role of the assistant surgeon / surgeon during patient positioning and a lack of standards concerning surgical positioning.

(2) Convert surgical instruments preparation from internal steps to external steps

As a countermeasure, the team could anticipate the preparation and counting of surgical instrument by preparing a sterile trolley in advance and by bringing them into the OR at the right moment. However, the current infrastructure does not feature a completely sterile area. This issue was identified by the team as an important element to be considered in new OR construction projects.

(3) Patient recovery should always occur in the recovery room

Although this theme may be obvious, the recovery room has limited space and is used for small procedures as well. Thus, patients stayed in the OR longer.

#### Improve Changeover Workflow

In terms of streamlining changeover workflow, the following themes emerged:

1. Need to create clear and shared standard work at inter-professional level: lack of standards emerged as a key reason for limited performances. Specifically, concerning standard work for the OR cleaning process, standard work for surgical instruments preparation, standard work on how to prepare patient before (i.e., patient positioning) and after surgery, standard work on how to restore ORs after surgery, standard role for the assistant surgeon, standards to organize spaces. Inter-professional collaboration strategies were encouraged by implementing as much as possible the culture of feedback, social interactions, multidisciplinary problem solving and open communication.
2. Importance of respecting OR planning and surgical cases scheduling: changes in the OR scheduling for the same day resulted in multiple phone calls within the OR and between the OR and the inpatient unit; furthermore, activities, materials, HPs and patients needed to be reorganized accordingly. Changes in the OR planning and scheduling impacts inevitably on the whole system.
3. Key role of the surgeon as process owner: the presence of the surgeon in the OR from the beginning and his or her active role in the preparation process would increase the performance of the entire system. The surgeon’s presence means that the surgery can be performed. If during changeover the team knew they would have to wait for the surgeon regardless of how quickly they performed, the general quality of their performance suffered.
4. Importance of improving changeover processes independently from surgery specialties: every OR has a dedicated team; however, as observed, changeover processes can occur between different specialties. Regardless of the surgery to be performed, changeover process times can and should be improved for all ORs independently from surgical cases, as the major steps are the same.

### Action Planning

While developing our action plan, goals were chosen to be clear, reachable, and with deadlines.

Based on the diagnosis phase, the team developed two kind of action plans:

The first action plan, *quick wins*, included general actions that would be implemented rapidly. For example, optimize nursing handover by reducing paper documents already available in the electronic patient system, or setting different times for the inpatient units to bring the first scheduled patient to avoid bottleneck and waiting times at the OR suite entrance. The quick wins actions emerged as countermeasures from the problems identified during the Gemba walks concerning the whole patient’s flow. The quick wins are available in Supplementary Table 1.

The second and main action plan was developed based on the SMED analysis. For each countermeasure (Table 3), key responsibilities and deadlines were defined. Countermeasures (i.e., the definition of new standards) were developed and tested by the inter-professional team, according to the responsibilities defined, during the daily practice The new protocols were then shared within the whole team and approved before implementation.

**TABLE 3:** Time invested in the project.

		Time (hours)*
1	Completion project charter: definition of preliminary goals, inter-professional team, project design	1.5
2	Training: SMED and gemba walks	1
3	Gemba walks in the OR suite and the inpatient units incl. video recordings	16
4	Transfer-session with the executive board	0.5
5	Video recordings analysis, gap analysis and action planning	8
	Implementation of action plan according to responsibilities	During the daily work
6	Status update on the implementation of the action plan (sharing new standard with the whole team)	3
7	Simulation in the OR	3
8	Training of the OR teams	During the daily work
9	Weekly management	Every monday (15 min.)
10	Evaluating – Gemba walks incl. video recordings	5
11	Transfer-session with the Executive board	1
12	Video recordings analysis and new countermeasures	2
13	Closing meeting	1
	Total hours	42

*The preparation time of the Project leader and the Lean facilitators as well as the time invested by the team in the development of the new standards is not included.

Based on the insight from the inter-professional team, the lean facilitators created and mapped an ideal process as a reference model for the simulation in the OR and to train-the-trainers (i.e., the team) on an inter-professional level. All new protocols were linked to the model as to define who has to do what and when. Times were defined based on non-complex surgeries. It can also be applied to every specific case (e.g., the team need to consider the timing according to the type of anesthesia). However, the defined protocols themselves do not change.

A complete list of all the new procedures is available in the Supplementary Table 2. The new protocols cover the following topics:

– Standard work for the OR cleaning process.
– Standard work for surgical instruments preparation.
– Standard work on how to prepare patient before and after surgery.
– Standard work on how to restore ORs after surgery.
– Role of the assistant surgeon as process owner.

### Taking Action and Evaluating

Actions and countermeasures to reduce changeover times were implemented from October to December 2020. Besides the countermeasures defined based on the SMED analysis, two further actions were undertaken. First, the changeover times were introduced as key performance indicators (KPI’s) at the OR *weekly management* huddle. The latter is a short standing inter-professional meeting during which the OR inter-professional management team evaluate KPIs and define the necessary countermeasures on a weekly basis, at a fix defined day and time. Second, surgery departments designated a resident surgeon fully dedicated to the OR during the day.

#### Impact on Changeover Times After One Month

The KPIs monitoring at the *weekly management* showed a positive impact on changeovers times. However, 1 month after the implementation, changeover times still showed a great variability for both specialties (min. 39 min, max. 66 min for gynecology; min. 51 min, max. 68 min for general surgery). Therefore, the inter-professional team conducted a second diagnosis round to understand what could be improved further.

#### Review – Second Diagnosis

Gemba walks were conducted again over the course of 1 day, and two changeover processes between three interventions were video recorded and analyzed. Changeover times lasted 47 min and 67 min. This variability reflected the variability reported during the *weekly management* huddle.

The following pitfalls emerged during this phase:

(1) Absence of the key role of the surgeon as process owner

In the first changeover, surgeons arrived when the patient was already positioned, and the patient needed to be re-positioned according to their indications. In the second changeover process, surgeons arrived on time, but did not contribute to the changeover process.

(2) Late start of induction procedure

In the second changeover, the type of anesthesia defined required more time than 15 min to be given, and the anesthesia team did not coordinate effectively to induct the patient in time. Identified causes for the delay were the last-minute change of the kind of surgery for the same patient as well as the kind of anesthesia required.

(3) Organization of the surgery inpatient unit: unit not ready to transfer the patient

A time frame of 31 min passed from the OR phone call until the patient arrived in the OR. In this time frame, there were multiple phone calls between the OR and the inpatient unit to understand why the patient had not yet been transferred.

(4) Changes in the or scheduling and planning

The OR scheduling was constantly changed, which consequently impacted the whole system and flows, including changeover processes. The particular context of the COVID-19 pandemic causes many changes because of the lack of bed available.

(5) Low implementation maturity and compliance to the new standards

Finally, the countermeasures were evaluated and the team agreed that although standards were defined and written, not all employees followed them. The analysis revealed the need to improve the training of all employees, to sustain the changeover process and to implement and define new working standard.

#### Impact on Changeover Times After Five Months

**FIGURE 3:**
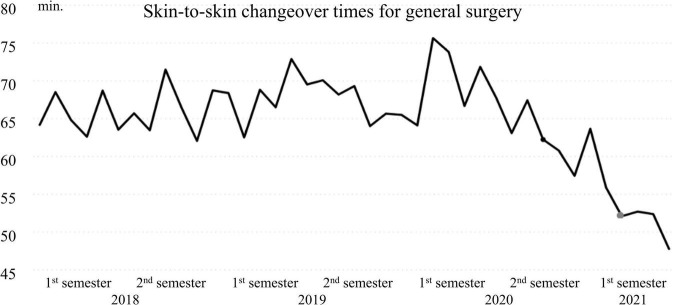
Mean changeover time (min.) per month from January 2018 to May 2021 for general surgery.

**FIGURE 4:**
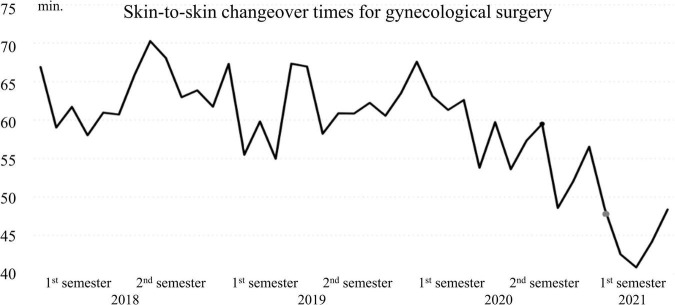
Mean changeover time (min.) per month from January 2018 to May 2021 for gynecological Surgery.

Historical data from 2018 to May 2021 showed a positive tendency of changeover times for both gynecological surgery and general surgery (Figures 3, 4). Performances improved significantly from 2020, specifically from October when the implementation of the countermeasures started.

Changeover times reached a new minimum (48 min for general Surgery and 41 min for gynecological surgery in March 2021, 3 months after the implementation). In comparison to 2019, the year before the current project, changeover times improved by 17 min on average for gynecology and 15 min for general. The primary objective of reducing changeover time by at least 25% was therefore achieved for both specialties.

## Discussion

This paper has highlighted how HPs were able to improve inter-professional collaboration by creating a common and shared process, and ultimately reduce changeover time between surgeries by 17 min for gynecology and 15 min for general surgery, on average.

### Fostering Inter-Professional Collaboration to Improve Processes and Quality of Care

The goal of the project was reached merely by improving inter-professional collaboration and enabling parallel processing. According to WHO, a “collaborative practice happens when multiple health workers from different professional backgrounds work together with patients, families, carers and communities to deliver the highest quality of care across settings” (26). However, inter-professional collaboration as a process does not happen by itself; it must be promoted and sustained at the different level of the organization. To achieve collaboration in this project, for instance, key actions were (a) defining and implementing standard work both on the clinical level (e.g., patient positioning) and on the organizational level (e.g.,. roles and responsibilities) by clarifying and writing what had to be done, how, when and by whom; (b) evaluating compliance to the new standard and providing further training; (c) constantly monitoring KPIs in the context of the weekly management system supported by the hospital Executive Board. Furthermore, the methodology of the project itself was a training for the team and a process to improve inter-professional collaboration. Applying both lean thinking on a macro-level and SMED on a micro-level is key to describe and replicate the methodology.

Improved inter-professional collaboration in turn enabled an effective parallel processing. Parallel processing occurs when a separate room is used for the induction and often require additional staff (18). Unlike similar projects conducted in other hospitals (15, 19), separate rooms for induction were already available (four induction room vs. five OR). However, as in the context of this project, the anesthesia team was responsible for consecutive surgeries, and therefore parallelizing induction with the OR process was initially difficult. To do so successfully without requiring additional staff, a higher level of coordination between the nurse and the doctor anesthetists as well as among all HPs is key.

The key actions emerging from this project have also been highlighted by the FOPH (27), who conducted the support program «Interprofessionality in healthcare 2017–2020» across different healthcare setting to improve inter-professional cooperation. Indeed, in one of its policy brief, the FOPH identified lack of clarity about tasks, competences and responsibilities among HPs as a barrier to inter-professional collaboration and thereby efficiency (28). Besides the need to better coordinate the different HPs roles, the FOPH highlighted the importance of constantly measuring and improving inter-professional collaboration and of institutionalizing inter-professional meeting such as huddles across the organization. In this, management plays a key role and must actively promote cultural change and the implementation of inter-professional collaboration at every level of the organization (28).

### Process Improvement Initiatives in the Context of a Comprehensive Management System

The considerations explained above are fundamental when planning a similar project in another hospital or setting. Both the methodology of the project and the context are key elements. The project was an improvement initiative promoted by the hospital executive board and the OR suite management, hence frontline HPs, who together discussed and negotiated together the goals to improve performances and quality.

Lean thinking offers a comprehensive management system (8, 21, 29) and a mindset that over time creates the hospital culture of continous improvement through waste elimination. This paper has showed that the integration of Lean techniques such as the SMED system, Gemba walks or huddles, are highly beneficial for the process of preparing, implementing and sustaining change. The project was a first step to promote such a comprehensive view of the patient journey and start the discussion on how the OR suite is performing as a system. The OR *weekly management* huddle includes thus other relevant KPIs that define how the whole OR performance is measured on time start (even if only for the first case (5), overrun time (time that exceeds the time scheduled per OR per day), OR occupancy rate, and prediction bias (bias in case duration). These KPIs are among the following eight interrelated KPIs for efficient OR management identified by Macario (30):

• Excess staffing cost in relation to productivity.
• Start-time tardiness for all elective cases per OR per day.
• Case cancelation rate the day of surgery.
• Postanesthesia care unit (PACU) admission delays (% of workdays with at least one delay in PACU admission).
• Contribution margin per OR hour.
• Turnover time (here defined as the interval Patient OUT – Patient IN).
• Prediction bias (bias in case duration estimates per 8 h of OR time).
• Prolonged changeover times (% of changeover that are more than 60 min).

Macario (30) highlighted the importance of respecting the OR scheduling as planned weeks to month ahead by pursuing the goal of getting the right case in the right room at the right time; this goal is a core principle of Lean Thinking.

### Convert Non-value-Adding Time of Changeover to Value-Adding Time of Procedures: Impact on Safety, Quality, and Costs

The implementation of Lean methodology allowed the hospital to reduce changeover time by 15 min and more for both specialties. Assuming an OR schedule for general surgery or gynecology with three surgeries (thus two changeovers), this would mean 30 min of time saved per room per day. Each OR minute is estimated to be worth 22–80 USD, as reported by Soliman et al. (31). Specifically, in the context of this project, one OR minute at EOC hospitals is estimated to be worth about 50 CHF, considering the costs of staffing, infrastructure, materials and medical devices. Thus, 30 min would represent an opportunity cost of 1500 CHF per room per day. This is to be considered in light of all the KPIs discussed previously which collectively impact on costs. The project allowed the hospital to free up resources – staff, infrastructure, devices - that could be employed differently, for example by anticipating surgeries and optimizing OR scheduling as to reduce overrun time. In general, the time recovered could serve both to reduce costs and increase revenue by increasing OR capacity and therefore allowing more surgeries to be performed. The actual impact on OR efficiency and costs must be calculated after extensive data collection on all surgical disciplines over a period of months. We are currently assessing such interesting aspects at our institution and we hope our future results will validate our thesis on applying Lean methodology to OR management.

However, besides money, it is important to consider to reinvest some of the time recovered on improved safety and quality. By reducing the amount of time spent on changeovers, HPs can spend more time providing higher quality of care to patients (5). Processes with defined standard work, that can be reviewed, continuously improved and audited anytime, allow to become safer and more robust in our practice inside the OR suite.

### Future Perspectives

Although the project focused on changeover time specifically, it opened a discussion on a broad range of topics that are critical when planning the OR of the future. Two themes in particular have emerged from these discussions. First, the analysis and the countermeasures demonstrated that different surgery specialties organize themselves independently. The project allowed to clearly define the role of the surgeons and when they are expected to be in the OR. This is fundamental to optimize changeover time and provides greater motivation for all OR staff (12). The gynecology department changed its organization to have an assistant surgeons fully dedicated to the OR activity during the day. Differently, in the general surgery department, the assistant surgeons worked both in the inpatient unit and in the OR during the same day. Thus, the question on how assistant surgeons should be organized – independently from the specialty – to better meet patient’s needs both in the OR and in the inpatient unit remains open. Surgeons often try to fit in other tasks between surgeries (32), and have been found to cause delays more frequently than any other profession in the OR environment (33). The need to fit in other tasks and the goal of reducing changeover time can thus be conflictual. Second, although changeover time in this hospital was optimized without changes in terms of infrastructure, the team highlighted several limitations concerning patient and instruments preparation due to lack of infrastructure that need to be considered in the future when planning and building a new OR suite. The methodology of this project, which includes Gemba walks, process mapping, simulation, and most of all the involvement of all stakeholder that are part of the process to be improved, provides insight on how to address construction project as well (forms follow functions). Innovative approaches to new OR planning and building such as Integrated Facility Design consider new construction to be an opportunity for transformation. This integrates all stakeholders (including patients’ and their families), who work closely together through the complex processes of OR management (9, 10, 34).

## Conclusion

Strong leadership and management commitment, proven methodology and system thinking approach are keys to improve inter-professional collaboration and reduce changeover time between surgeries at low cost. The AR project allowed the hospital to reach the goal of reducing changeover times for gynecological and general surgery at least by 25% on average, without changing infrastructure, technology or other resources. For other healthcare facilities that wish to make changeover more efficient and obtain sustainable results over time, of most importance is to define a common goal between all stakeholders, define and implement standard work that regulate roles, responsibilities and tasks of all stakeholders involved in the process, define and execute parallel processing, by converting changeover steps from internal to external, introduce and/or reinforce the role of the surgeon as process owner, optimize OR planning and scheduling. Finally, the support of the hospital executive board is fundamental for the successful, sustainable implementation of changes and continuous improvement over time.

## Data Availability Statement

The raw data supporting the conclusions of this article will be made available by the authors, without undue reservation.

## Ethics Statement

Ethical review and approval was not required for the study on human participants in accordance with the local legislation and institutional requirements. Written informed consent for participation was not required for this study in accordance with the national legislation and the institutional requirements.

## Author Contributions

AS, MA, AB, AV, and DL: protocol and project development. AV, CT, AL, MA, and FM: data acquisition and interpretation. FM: statistical analysis. MA, AV, CT, and AL: manuscript drafting. AS, FM, AB, AV, and DL: manuscript revision and accountable for all aspects of the work. All authors approved the final version of the manuscript.

## Conflict of Interest

The authors declare that the research was conducted in the absence of any commercial or financial relationships that could be construed as a potential conflict of interest.

## Publisher’s Note

All claims expressed in this article are solely those of the authors and do not necessarily represent those of their affiliated organizations, or those of the publisher, the editors and the reviewers. Any product that may be evaluated in this article, or claim that may be made by its manufacturer, is not guaranteed or endorsed by the publisher.
