# Supplementary material for: Reducing Changeover Time Between Surgeries Through Lean Thinking: An Action Research Project
Source: Front Med (Lausanne). 2022 Apr 27;9:822964. doi: 10.3389/fmed.2022.822964 (PMC9091348; doi:10.3389/fmed.2022.822964)
Supplement: Supplementary file 1 [file Table_4.docx]

Supplementary Table 1. Quick Wins

| **Problems observed** | **Suggested countermeasures** |
| --- | --- |
| Long patient waiting times outside the OR entrance (in the corridor of the surgery inpatient unit). It is required that all patients be there at 7:15 AM, and this creates bottlenecks. | Define different times (e.g., 7:15 for two wards and 7:20 for the other two wards). Also define whether the patient should be at the OR entrance or in front of the recovery room. |
| Double handover: reading the patient’s medical dossier and talking to the inpatient unit nurse about the same information. | Define a standard for the nurse handover regarding which documents need to be handed over on paper (not available in the electronic information system). Transfers of patients from the wards to the OR could then be performed by other professionals (except in special situations). |
| Operating table not ready when the patient arrives. | When the OR is ready, the nurse anesthetist should inform both the ward and the person responsible to bring the patient into the OR suite. This procedure is already defined and it is therefore important to understand why it is not happening. |
| Type of anesthesia required not reported in the electronic information system. Furthermore, the prescription of antibiotics is missing in the system; thus, the nurse needs to verify with the surgeon if it was forgotten or if the antibiotic is not necessary. | Record the kind of anesthesia in the electronic information system (from the pre-surgery assessment). Insert a mandatory field in the electronic information system (yes/no) to indicate if the antibiotic is necessary. |
| Many calls from the OR to the inpatient units. | Define when to inform the inpatient units so they know when to prepare patients and have time to organize the transfer. |
| The OR calls the inpatient units to communicate that they should start pre-medication for the patient, even if it is not prescribed (and thus unnecessary). | Inform the inpatient units only if there is a prescription in the electronic information system. |
| Last-minute changes in OR planning: the inpatient units (HPs and patients) are not informed and do not know when surgeries are postponed. | Inform the inpatient units in case of changes and provide them with information concerning the new scheduling and pre-operative fasting criteria. |
| Lack of coordination during the OR cleaning process. | Define a clear procedure on how to perform the process with one housekeeper and with two housekeepers. |
| Foam must always be used to clean surgical instruments to avoid damage; this is not being done. | Implement increased controls. |

Supplementary Table 2. List of new protocols

| **Number** | **Protocol** |
| --- | --- |
| I-SOP-066 | Procedures between surgeries  *Surgical technicians’ task (sterile): in the preparation phase and in the final phase of surgery*  *Surgical technicians’ task (support): in the preparation phase and in the final phase of surgery*  *Role standard for the anesthesia team* |
| I-SOP-065 | Call to the OR mode and timing – Role standard for the assistant surgeon in the OR |
| I-ALB-053 | OR cleaning procedures between surgeries |
| I-ANES-167 | Patient privacy from the beginning to the end of surgery |
| **Standard operating procedure – gynecological patient positionings** | |
| I-SOP-5569  I-SOP-5570  I-SOP-5571  I-SOP-5572  I-SOP-5573  I-SOP-5574  I-SOP-5575  I-SOP-5576 | Breast  Caesarean section  Gynecological laparotomy  Vaginal hysterectomy  Hysteroscopy  Gynecological simple procedure  Laparoscopic hysterectomy  Laparoscopy |
| **Standard operating procedure – general surgery patient positionings**  *Laparotomy surgery (LT)* | |
| I-SOP-2061  I-SOP-2062  I-SOP-2063  I-SOP-2064  I-SOP-2065  I-SOP-2066  I-SOP-2067  I-SOP-5577  I-SOP-2074 | Exploratory laparotomy  Thyroidectomy LT  Sigmoidectomy LT  Cholecystectomy LT & surgical revision of biliary strictures  Hemicolectomy (right) LT  Hernioplasty LT  Ileostomy & colostomy LT  Hemorrhoidectomy LT  Sacral dermoid LT |
| *Laparoscopic surgery (LS)* | |
| I-SOP-2068  I-SOP-2069  I-SOP-2070  I-SOP-2071  I-SOP-2072  I-SOP-2073 | Cholecystectomy LS  Inguinal hernia LS  Umbilical hernia & epigastric hernia LS  Sigmoidectomy LS  Gastrectomy & fundoplication LS  Appendectomy LS |
